# Understanding Tendons: Lessons from Transgenic Mouse Models

**DOI:** 10.1089/scd.2018.0121

**Published:** 2018-09-01

**Authors:** Manuel Delgado Caceres, Christian G. Pfeifer, Denitsa Docheva

**Affiliations:** ^1^ Experimental Trauma Surgery, Department of Trauma Surgery, University Regensburg Medical Centre, Regensburg, Germany.; ^2^ Department of Trauma Surgery, University Regensburg Medical Centre, Regensburg, Germany.; ^3^ Department of Medical Biology, Medical University-Plovdiv, Plovdiv, Bulgaria.

**Keywords:** tendon and ligaments, tendon biology, mice models, knockout mice, transgenic technology, tendon phenotype

## Abstract

Tendons and ligaments are connective tissues that have been comparatively less studied than muscle and cartilage/bone, even though they are crucial for proper function of the musculoskeletal system. In tendon biology, considerable progress has been made in identifying tendon-specific genes (Scleraxis, Mohawk, and Tenomodulin) in the past decade. However, besides tendon function and the knowledge of a small number of important players in tendon biology, neither the ontogeny of the tenogenic lineage nor signaling cascades have been fully understood. This results in major drawbacks in treatment and repair options following tendon degeneration. In this review, we have systematically evaluated publications describing tendon-related genes, which were studied in depth and characterized by using knockout technologies and the subsequently generated transgenic mouse models (Tg) (knockout mice, KO). We report in a tabular manner, that from a total of 24 tendon-related genes, in 22 of the respective knockout mouse models, phenotypic changes were detected. Additionally, in some of the models it was described at which developmental stages these changes appeared and progressed. To summarize, only loss of Scleraxis and TGFβ signaling led to severe tendon developmental phenotypes, while mice deficient for various proteoglycans, Mohawk, EGR1 and 2, and Tenomodulin presented mild phenotypes. These data suggest that the tendon developmental system is well organized, orchestrated, and backed up; this is even more evident among the members of the proteoglycan family, where the compensatory effects are much clearer. In future, it will be of great importance to discover additional master tendon transcription factors and the genes that play crucial roles in tendon development. This would improve our understanding of the genetic makeup of tendons, and will increase the chances of generating tendon-specific drugs to advance overall treatment strategies.

## Introduction

### Tendon development, critical factors and signaling cascades

The establishment of a proper musculoskeletal system involves the finely orchestrated development of muscle, cartilage, and tendon lineages emerging from the somitic mesoderm [1]. During embryonic development, muscle and cartilage (systems that are better understood and studied than tendon) arise from the myotome and sclerotome respectively, in response to signals emitted from neighboring tissues. The tendon lineage is formed within the dorsolateral sclerotome, adjacent to and beneath the myotome, in a somite subdomain denominated as syndetome [1]. The ontogeny of the tenogenic lineage is not fully understood yet because of the absence of specific early tendon lineage markers.

To our knowledge, there are two stages related to tendon development. First, the emergence of precursors/progenitors based on their origin and localization and second, commitment and differentiation based on pivotal signaling cascades. With the identification of the beta helix-loop-helix transcription factor Scleraxis (Scx), an important and distinctive marker for early tendon development was found [2,3]. Schweitzer et al. reported the usage of Scx expression as a method for the identification of a pool of tendon progenitors in the mesenchyme subjacent to the ectoderm [3]. In mice, embryonic tendon and ligament development starts occurring between E9.5–E12.5 [4].

The main function of the axial tendon is to bond the muscles that are located along the spinal column to the vertebrae and transfer the generated force to the axial skeleton, providing spinal stability and range of movement [4]. Axial tendon progenitors, which per definition are Scx-expressing cells, originate during embryonic development from the ventral compartment of emerging somites, more precisely, from the syndetome, one of the four somatic sub compartments [5–8].

**FIG. 1.**
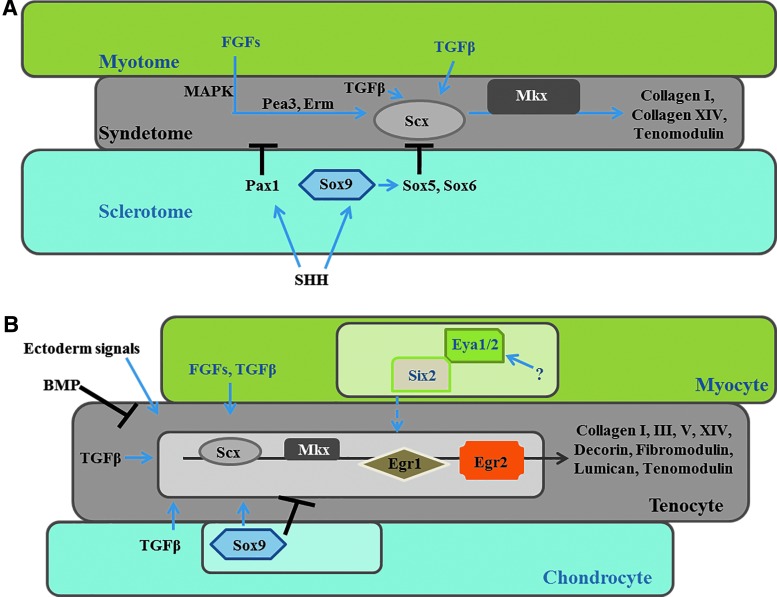
Development of axial and limb tendons during embryogenesis. **(A)** Axial tendon differentiation starts with upcoming FGF signaling from myotome. Signals from the sclerotome, for example, Sox9 (activated by SHH) have a negative effect on Scx induction blocking its expression. Moreover, TGFβ signaling influences Scx and Mkx expression promoting axial tendon differentiation and the activation of extracellular matrix proteins such as collagen I, collagen XIV, tenomodulin, and others. **(B)** Limb tendons are formed differently compared to axial tendons. Tendon limb progenitors are induced by ectodermal signals in the limbs and inhibited by BMP. Tendon progenitors position themselves between differentiating muscles and cartilage, condensate, and differentiate to form proper tendon tissue. Not only FGF but also TGFβ can induce limb tendons. Six2 is highly expressed in forming limb tendon cells, but the role of Eya1/2 is still controversial (therefore indicated with?). As in the axial tendon development, Scx and Mkx play a pivotal role giving the starting impulse for limb tendon formation. Early growth response 1 and 2 (Egr1/2) transcription factors act as molecular sensors for mechanical signals guiding the final steps of tendon maturation and production of collagen I, III, V, XIV, proteoglycans (decorin, fibromodulin, lumican), and tenomodulin. Figure was adapted from [10]. BMP, bone morphogenetic protein; FGF, fibroblast growth factors; SHH, sonic hedgehog.

Fibroblast growth factors (FGF) play an important role in chick and mouse embryos during axial tendon development (Fig. 1A). In mice, FGF signaling starts from the upper myotome resulting in the activation of the mitogen-activated protein kinase pathway, E26 transformation-specific sequence (Ets) transcription factors, Phosphatidylinositol-4-phosphate 5-kinase (Pea3) and Ezrin/radixin/moesin (Erm). Lastly, Scx and transcription factor Mohawk (Mkx) promote final tendon lineage commitment and differentiation; this is characterized by the expression of collagen type I, type XIV, and tenomodulin (Tnmd) [1,9,10]; Tnmd is to date, the best-known mature marker for tendons [11–13].

On the other side, ventral midline sonic hedgehog expression can solely activate Pax1, which consequently has a negative effect on Scx, hindering its induction in the sclerotome [10]. While signals coming from the myotome positively influence axial tendon development, signals derived from the sclerotome have an adverse effect. This starts with the expression of Sox9 and subsequent induction of Sox5 and Sox6, which block Scx expression [10].

Progenitors of the limb tendons develop differently to the cells that give rise to axial tendons because they are not localized within a specific subdomain in the somite but rather around the lateral plate mesoderm [7,14]. Limb tendon progenitor cells are intermingled with migrating myoblasts in ventral and dorsal parts of the limb bud [8,15]. During limb development, Scx-positive tendon progenitors are induced by ectodermal signals and constrained by bone morphogenetic protein [10] in the limbs and position themselves between differentiating muscles and cartilage. Subsequently, they condense and finally differentiate to form distinct tendons [8] (Fig. 1B).

Not only FGF signaling has a pivotal role in regulating limb tendon differentiation, proteins from the TGFβ superfamily have been reported to be inducers of limb bud tendons [16–19] (Fig. 1B). It has been previously described that the disruption of TGFβ signal in double mutant *Tgfβ2^−/−^/Tgfβ3^−/−^* mouse embryos leads to the loss of most tendons and ligaments in the limbs, trunk, tail, and head [20].

The close interaction between muscles and tendons during limb development [7,15,21] suggests that transcription factors such as Six homeobox 1 and 2 (Six1/2) and EYA transcriptional coactivator and phosphatase 1 and 2 (Eya1/2) [22] might play an indirect role in tendon formation. However, Bonnin et al. showed using *Six1*-deficient mice that this gene is neither expressed in tendons nor essential for tendon development [23]. Whole transcriptome expression profiling of mouse limb tendons using RNA-Seq. revealed no differential expression of *Six1*, while *Six2* was found to be highly expressed in forming limb tendon cells at E13.5 [24].

As in axial tendon development, Scx and Mkx play a crucial role, giving the initial impulse for limb tendon formation. In a second step, Early growth response 1 and 2 (Egr1/2) act as molecular sensors for mechanical signals [25] guiding and regulating collagen maturation and leading to final tendon differentiation [10,26–28].

The exact mechanisms triggering tenogenesis, which need to be finely orchestrated to guide progenitor cells to fully differentiated tendon cells, still remain elusive and worth further investigation since the whole process is dependent on various factors with different signaling cascades amongst the diverse cellular compartments.

### Tendon function, composition, and structure

Tendons appear to be simply organized tissues with the main function of connecting muscles with bones transmitting the muscle-generated force, thus allowing joint movements [29,30]. Tendons, which could be compared to flexible strings, are primarily made of collagens and a minor fraction of elastin surrounded by a proteoglycan-rich matrix. Concerning the mechanical function, tendons can be classified as positional tendons, which are primarily loaded along their long axis permitting the interplay between muscles and bones [29,31] and energy storing tendons that are more elastic and extensible and when loaded, release the accumulated energy to improve the efficiency of movement [32].

The tendon extracellular matrix consists mainly of collagens (60–85% of tissue's dry weight) [33], with type I being the most prominent one (∼95%) and small amounts of type V, VI, XII, XIV, and XV [34,35]. The role of collagens within tendons has been well studied and characterized [36–38] but the noncollagenous part of the matrix consisting of proteoglycans like fibromodulin, lumican, biglycan, and decorin [39–41] requires more investigation. Recent advances in proteoglycans research will be discussed later.

Tendons are hierarchically organized structures with collagen fibrils as the smallest structural units; these are composed of parallel chains of collagen molecules bound together by covalent cross-links [30]. Fibrils aggregate gradually with other fibrils to form tube-like structures called fibers, which subsequently attach to other fibers and arrange themselves into bundles (collagen-rich fascicles). Collagen fascicles are aligned in the direction of force application [42] and are surrounded by thin layers of connective tissue known as endo- and epitenon (tendon sheets), where nerves, blood vessels, and tendon stem/progenitor cells are situated [41]. Fully differentiated tendon cells (tenocytes) are localized between the collagen fibers [41].

## Power of Knockout Technologies

Knockout/-in technologies are a powerful and indispensable tool to study gene functions in vivo. In 2007, the Nobel Committee for Physiology or Medicine awarded Drs. Capecchi, Evans, and Smithies with the Nobel Prize for their discoveries in the field. The identification and isolation of stem cells of the early mouse embryo, the proper cell culture conditions, and the reintroduction of the genetically modified cells into foster mice mothers was established by Martin J. Evans and Matthew Kaufmann in the early 80s [43]. Mario Capecchi and Oliver Smithies, working independently of each other, discovered the mechanism behind homologous recombination [44,45], creating the fundamentals for the production of the first knockout mice [46,47].

Due to the startling gene homology between mice and humans (99%) [48], mouse knockout technology has great advantages for the understanding of human biology and disease [48–50]. The mouse, as an experimental model is fundamental in biomedical research, since its development, physiology, behavior, and diseases are similar to those in humans [49]. Additionally, mouse properties such as short life spans and high reproduction rates make it very suitable for low-cost genetic studies.

The most obvious approach to investigate a gene's function is by generating a knockout and to validate the outcome by analyzing the whole animal phenotype [51]. The capacity to manipulate and introduce specific DNA sequences (eg, genes) has been used for biomedical purposes for more than three decades [52]. Furthermore, with the introduction of CRISPR/Cas-9 and CRISPR/Cas-13 technologies, new and precise tools for genome engineering are now available [53,54].

There are two main methods for the generation of knockout mice: (i) Gene trapping [55,56] and (ii) gene targeting [43,45]. Gene trapping mutagenesis was first developed for the detection of different gene expression patterns [57] but it is now well established as random mutation technique. Gene targeting is based on homologous recombination and proper manipulation of embryonic stem cells (ES cells). This technique permits the insertion of genetic mutations into a mouse at a specific genomic locus and generates full gene knockout, point mutation, deletion/insertion (indel), and others. [58]. In this review we will shortly describe options and techniques for the generation of mouse knockout models.

### Conventional knockout mouse model (constitutive)

**FIG. 2.**
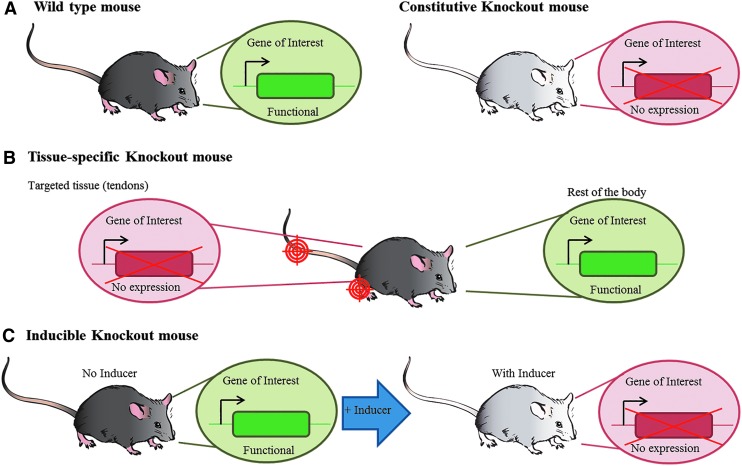
Schematic representation of constitutive, conditional, and inducible knockout mouse models. **(A)** In a wild-type mouse, the GOI is functional. In a constitutive knockout mouse the GOI is not functional and therefore not expressed. The generated Tg-mouse might show phenotypic changes, depending on which gene has been knocked out. **(B)** In a tissue-specific knockout mouse the GOI is not functional in a specific tissue, while normal gene functionality is detected in the rest of the organism. In the cartoon the *red* targeted area illustrates that the gene is inactivated only in tendon tissues such as in the tail and Achilles tendons. **(C)** Inducible knockout systems allow the inactivation of a GOI by the addition of an inducer (in defined doses) at a given time point and within a specific tissue, before that, gene of interest is functional. Figure was adapted from [154–156]. GOI, gene of interest

This biological model targets a gene, which will be constitutively inactivated and has therefore a major effect at all stages of development (Fig. 2A). The method is based on the inactivation of the gene of interest by the insertion of a null mutation into an essential coding region in a specific genetic locus [58]. The modified sequence is inserted into a targeting vector containing an antibiotic resistance gene between the homologous regions (eg, neomycin). This step will allow a subsequent selection for positively transfected ES cells [58–60].

The technique commonly used for transfection is electroporation, because of its high efficiency and stable DNA introduction [61]. ES cells containing the mutagenized DNA construct are inserted into blastocysts, which are finally transferred into pseudopregnant females for the production of chimeric mice [60]. Offspring from chimeric mice have to be screened for targeted mutation and inbred to generate homozygous knockout mice. The main disadvantage of a constitutive knockout system is the fact, that roughly one-third of the genes present in mammals (∼8,000 genes in mice) are essential. This means that the deletion of two copies of some genes may either lead to embryonic or postnatal lethality, or could activate a nonregulated expression of other compensatory genes [62].

### Tissue-specific knockout mice (conditional)

An interesting and effective approach to circumvent embryonic lethality or compensatory mechanisms, is the generation of conditional knockout mice by the use of, for example, Cre/loxP- (Cre recombinase/loxP sites) [63] or Flp/FRT technology (Flp recombinase/FRT sites) [64,65]. A target gene can be specifically inactivated in defined tissue(s), while its expression stays unaffected in the rest of the body [58] (Fig. 2B). Of great benefit is the possibility to inactivate a particular gene in a predefined cell lineage or at certain stage of development (eg, in the tenogenic lineage, *Scx*) [66].

For the proper usage of the Cre-loxP recombination system two mouse strains are required: the first one, called the deleter mouse line (where the Cre-recombinase is expressed) and the second strain carries the targeted gene flanked by two loxP sites. When intercrossed, the resulting offspring carries both, Cre-recombinase and loxP-flanked (“floxed”) target gene. In the cells, where the *Cre* gene is expressed and due to Cre-loxP site-dependent recombination deletion/excision of targeted gene will occur [67].

The main disadvantage of this approach is the time taken to develop this model, since there is the need for an additional breeding steps with the tissue-specific deleter mouse line. Additionally, it has been reported that the efficiency of gene deletion differs depending not only on the age and sex of the transgenic animal but also on gene locus position and Cre activity [68,69].

### Inducible knockout mouse models

Inducible transgenic mouse models allow the activation/inactivation of a target gene at a given time point and within a particular tissue. Before this, the gene of interest is either expressed or not (Fig. 2C). The first inducible system, based on tetracycline, called Tet-off and Tet-on system, was developed by Gossen and Bujard [70,71]. Other systems were later developed, including the tamoxifen system [72].

The inducible gene expression regulation is based on a bi-transgenic system and the estrogen receptor (ER) ligand-binding domain [73,74]. Transcription factors or recombinases can be genetically modified and subsequently be ligated into the mutated ligand-binding domain of the ER. Therefore, genes can be turned/switched on and off by administering triggering compounds [74]. The main advantage is that inactivation can be done in all cells of the body or in a tissue-specific manner. Conversely, the main disadvantage relays in the important role played by the inducer since it may generate complications in the phenotype.

### CRISPR-Cas technology

This new technology takes advantage of precise genome engineering tools to increase the feasibility for the faster generation of transgenic mice models, which is of great importance for more in-depth study of the musculoskeletal system. Nelson et al. showed that in vivo genome editing improved muscle function in a Duchenne muscular dystrophy mouse model; Tabebordbar et al. and Suzuki et al. have also shown similar results [75–77]. The accessibility to this technology has increased rapidly in the last years and the simplicity of the system is its greatest advantage. Naturally, there are important issues that have to be taken into account while working with this tool [78].

The classical approach for the generation of knockouts is by injecting Cas9 mRNA and the single guide(s) RNA (sgRNA) directly into mouse embryos producing accurate edits into specific genomic loci. Mice that develop from the modified embryos have to be sequenced and/or genotyped to confirm that they carry the wanted mutation and bred to corroborate that a germline transmission is possible. The offspring can directly be crossed saving time and resources and eliminating the necessity of backcrossing.

## Translation and Relevance for Clinical Treatment

To unveil critical mechanisms in tissue degeneration and regeneration, knockout or knock-in animal models of specific genes is of utmost importance. Small animals seem to be genetically very similar to human beings, a high number of genetic patterns and downstream mechanisms are comparable and preserved throughout evolution. Thus, genetically altered mouse models have already been used in the musculoskeletal field to uncover diseases and develop potential therapies for clinical use. For example, fibrodysplasia ossificans progressiva (FOP) is caused by a genetic variant in the glycine/serine activation domain of activing A type I receptor/activin-like kinase 2 (ACVR1/ALK2) and leads to lethal ossifications of soft tissues in the developing and growing human body [79,80].

This gene mutation was mirrored in a knock-in mouse, which developed FOP and is therefore, now used for generation of specific therapies against this lethal disease [81]. Most recently, it was shown that depletion of mast cells in these mice leads to less severe heterotopic ossifications and minor dysplastic bone formation compared with untreated animals, paving the way for clinical translation [82]. As this example from bone formation disorder shows, genetically altered small animals are of great use for increasing insights into clinically relevant illnesses and may serve as a starting point for the development of clinically useful therapies.

A knockout mouse model of biglycan and fibromodulin, both of which are important contributors to tendon development and tendinous homeostasis, has shown heterotopic ossification of tendons [83]. Biglycan is a X-chromosome related small leucine-rich proteoglycan and due to its genetic location is underexpressed in X-chromosome-deficient patients and overexpressed in multi-X-chromosome aberrations, such as Turner syndrome (X0) and Klinefelter syndrome (XX+) respectively. While Turner's syndrome is characterized by short stature, Klinefelter syndrome is characterized by tall stature [84].

However, single knockouts of biglycan only led to mild age-related osteopenia due to the lack of osteogenetic precursors [85]. Ultrastructural analysis of singly biglycan knockout mice showed disorganization of the collagen network in structural tissues, mimicking Ehlers-Danlos-like changes [86]. Interestingly, decorin (Dcn) was upregulated in these knockout mice, suggesting a compensation of biglycan loss on a molecular level [87] and further studies using a double biglycan/decorin knockout revealed severe osteopenia, overall reduced health status and infertility [86]. Furthermore, it was shown, that severity of tendon calcification is dependent on single vs. double knockout, age and biglycan vs. fibromodulin knockout [88].

Working groups studying tendon biology have suggested that, based on published data, tendon degeneration, growth, and regeneration have underlying multifactorial genetic contributors [85–89]. To gain insight in different genetic regulators and correlate those with clinical findings, both small animal gene knockout and clinically relevant studies have to be conducted. As stated before, experimental studies using transgenic animals and different models, such as functional performance [13], injury [90], and inflammation/degeneration [91] should be stressed out.

An example of relevant experimental knockout studies was published by Dex et al., where the endurance running performance of *Tenomodulin*-deficient (*Tnmd*-KO) mice was analyzed [13]. *Tnmd*-KO mice have been previously described and showed a mild tendon but no developmental phenotype [11]. *Tnmd* deficiency led to significantly inferior running performance that worsened with training.

Using the same transgenic line, Lin et al. applied an Achilles tendon injury model to challenge the role of Tnmd in early tendon healing [90]. Detailed analysis showed a very different scar organization in the injury site of the KO tendons with an augmented adipocyte and blood vessel accumulation. This demonstrated that Tnmd is needed to prevent fat cells accumulation and fibrovascular scar formation, which subsequently have an impact in tendon functionality and healing [90].

There are three different scenarios that might help to optimize the study and translation of animal models to the development of future clinical treatments. (i) Target a specific gene to see whether the generated phenotype mimics a human disease; (ii) find a human disease with the respective genetic mutation and generate a KO-mouse model to understand the mechanisms involved, and (iii) use transgenic mice with no developmental phenotype and challenge the system testing the above-mentioned models (performance, injury, and inflammation/degeneration).

Thus, genetic animal models will serve in the future to unveil contributors for tendon disorders and add to the development of clinically meaningful therapies whether in prevention of tendon degeneration and injuries or improving the speed and effectiveness of recovery of tendon ruptures.

## Genes Involved in Tendon Development

Over the past 30 years, the usage of transgenic technologies has proved to be a very powerful and helpful tool for in-depth gene studies, not only by “loss-of-function” experiments but also by targeting a specific gene's overexpression. New tools and methods have supported a more detailed molecular analysis of tendons including the commitment, development, maturation, and consolidation of progenitor cells into fully differentiated tendon cells.

We have screened for a large number of tendon-related genes, which have been previously studied using corresponding gene-deficient mice (Table 1). We have summarized and classified diverse transgenic mice strains that have been used to analyze specific gene's function and have been of great help to understand tendon biology (Table 2). Moreover, we have described meticulously, if a phenotype is evident in constitutive, conditional, or inducible knockout mice, especially regarding tendon tissue morphology, structure, or mechanical function. One of the first descriptions made in the table is the time point by which the phenotype is noticeable; we specify how the tissue affected is organized, which changes are present at a cellular and ultra-microscopic level, and whether the biomechanics of the tendons were compromised.

**Table 1.** List of Genes Involved in Tendon Development

*Gene*	*Category*	*Phenotype in gene targeted mice*	*Phenotype in human*	*Reference*
Biglycan (*Bgn*)	Proteoglycan	During development. Effect is age dependent. Deficient mice developed ectopic tendons and joint ossification, and osteoarthritis 3 months after birth. Injured Bgn^−/−^ tendons are more cellular than WT. In *Bgn/Fmod* double null mice, prevented formation of mature collagen fibrils. Weaker tendons with decreased stiffness. Alteration in collagen fibril structure, larger diameter, and abnormal morphology. Altered mech. properties.	Associated with Turner and Klinefelter syndrome.	[84,96–101]
Bone morphogenetic protein (*Bmp4*)	Growth factor	Enthesis formation inhibited. Failure in bone ridge formation.	None reported	[102,103]
Collagen type I (*Col1a1, Col1a2*)	Protein	*Col 1^−/−^* mutant mice died at E12.5 due to vascular defect (rupture of major blood vessels). In *Col1a1^(*Jrt*)/+^* mice, collagen fibrils are smaller in diameter compared to WT. Tail tendons are frayed.	Ehlers-Danlos syndromes with joint hypermobility, skin hyperlaxity, and hyperextensibility, osteogenesis imperfecta, bone fragility, blue sclera, dentinogenesis imperfecta.	[104–109]
Cartilage oligomeric matrix protein (*Comp*)	Glycoprotein	No morphological abnormalities in *Comp^−/−^* mice sternum, vertebra, or tendons in tail. Achilles and tail tendons showed normal collagen fibrillary network in ECM.	Multiple epiphyseal dysplasia or pseudo- achondroplasia. Severe short-limb dwarfism and early onset osteoarthritis.	[110–112]
Decorin (*Dcn*)	Proteoglycan	Effect is observable at all stages of tendon development. Dysfunctional regulation of fibril assembly. Collagen fibrils are coarse, irregular, and haphazardly arranged. Individual fibrils showed irregular profiles and abnormal lateral association with adjacent fibrils. Tendons possess greater viscous properties. Significant reduction in collagen content. Periodontal ligament (PDL) showed hypercellularity in *Dcn^−/−^* mice. Tendons present larger fibril diameters. Tendons from mature deficient mice have significantly reduced strength, maximum load and stress, stiffness, and modulus.	Associated with Ehlers-Danlos syndromes	[98,99,101,105,113–115]
Early growth response factor 1 and 2 (*Egr1 & 2*)	Transcription factor	Tendon phenotype starting at E18.5. *Egr1^−/−^* mice present lower number of individual tail tendons and diminution in the diameter. Reduced expression of Scx, Mkx, and Tnmd. Decreased number of collagen type I fibrils. *Egr1^−/−^* mice tendons were mechanically weaker and more fragile compared to WT littermates.	None reported	[10,27,28]
Estrogen model	Steroid hormone	In rabbit model, reduction of blood estrogen level is associated with reduction in tensile strength, decrease in collagen synthesis, fiber diameter, and density and increase degradation in tendon tissue.	None reported	[116]
Fibrillin 1 and 2 (*Fbn 1 & 2*)	Proteoglycan	Postnatal stage. Flexor digitorum longus tendons from *Fbn2*- null mice showed decreased collagen cross-links. No alteration in total collagen content. No structural differences observable in collagen fibril diameter, size, or number. *Fbn1*-deficient tendons showed loss of tensile strength.	Marphan syndrome (Fbn1 mutation). Beals syndrome, a.k.a congenital contractural arachnodactyly (CCA) (Fbn2 mutation)	[117,118]
Fibromodulin (*Fbn*)	Proteoglycan	Small diameter and immature collagen fibrils without progression to mature large diameter fibrils. Thinner collagen fibers. Increased content of noncross-linked Col1a2. Reduced number of cells in tail tendons. Compensatory increase of Lumican. Achilles tendon from *Fmod^−/−^* mice showed collagen fibrils with irregular and rough outlines in cross section. Reduced tendon stiffness. Increased tendon flexibility and impaired tendon function, meaning loss of tendon strength.	High myopia	[101,105,119–122]
Growth differentiation factor-5 (*Gdf-5*)	Growth factor	Postnatal stage. *Gdf-5^−/−^* mice have severe limb shortening and joint defects (dislocation). Tendons of *Gdf-5^−/−^* mice are smaller and display decreased Col1 content. Increased frequency toward small diameter and irregularly shaped collagen fibrils. Decreased peak stress, stiffness, and energy absorption.	Acromesomelic chondrodysplasia, Hunter-Thompson type.	[123–125]
Integrin beta-1 (*Itg-β1*)	Cell surface receptor Transmembrane protein	Mice deficient in *Itgβ1* died shortly after implantation. Peri-implantation lethality is characterized by inner mass failure.	None reported	[126,127]
Integrin alpha-11 (*Itg-α11*)	Cell surface receptor Transmembrane protein	*Itgα11*-deficient mouse displayed dwarfism with increased mortality (starting at 1 year of age) due to disorganized periodontal ligaments and defective incisors. Incisor PDL showed augmented thickness and increased collagen accumulation.	None reported	[128,129]
Lumican (*Lum*)	Proteoglycan	Earlier stage of development (postnatal, 2–14 days). Large-diameter collagen fibrils form disorganized matrix. No compensatory increase in Fmod. *Lum^−/−^/Fmod^−/−^* mice show collagen fibrils with cauliflower-like contours, suggesting atypical lateral growth in fibrils. No loss in tendon biomechanical function.	High myopia	[101,105,119–121]
Myostatin (*Mstn*)	Growth factor	Mstn plays a role in both, prenatal and postnatal development and regulation of tendons. Deficient mice have small and brittle tendons. Decreased Col1, Scx, and Tnmd expression. Hypocellular tendons with decreased fibroblast density. Tendons present high peak stress, low peak strain, and increased stiffness.	None reported	[123,130]
Mohawk (*Mkx*)	Transcription factor	Starting at E16.5. Decreased expression of Col1, Fmod, and Tnmd. In postnatal stages, tendon sheaths are thicker and contain more cell layers. Three-month old animals have hypoplastic, smaller, and less vibrant tendons throughout the body. Reduction in tendon mass and tendon thickness. *Mkx^−/−^* exhibit a wavy-tail phenotype. Diminution of tensile strength in Mkx null Achilles tendons.	None reported	[10,131–134]
Scleraxis (*Scx*)	Transcription factor	Starting at E13.5. Null mice display reduced and disorganized tendon matrix. Severe disruption of tendon formation resulting in tendon defect (tendon progenitors fail to condense into morphologically distinct tendons). Mutant tendons are hypocellular. Endotenon cells appeared disorganized and failed to generate a continuous layer, resulting in the loss of structural integrity. Microfibrils in mutant tendons are highly disorganized. *Scx^−/−^* mice have impaired locomotion and a complete inability to use their tails. The force-transmitting tendons are severely disrupted but ligament and short-range anchoring tendons are not affected	None reported	[10,123, 135–137]
Tenomodulin (*Tnmd*)	Glycoprotein	Postnatal. One-month old *Tnmd*-deficient mice showed decreased tenocyte proliferation and density. Six-month-old mutant displayed reduced cell numbers (hypocellular). Cross sections of tendons from *Tnmd^−/−^* mice showed variations in collagen fibril distribution and overall thicker collagen fibers. Fibrillar surfaces are uneven and rough.	SNPs associated with: obesity, type 2 diabetes, age-related macular degeneration, APOE, and Alzheimer's disease	[11–13,41,138,139]
Transforming growth factor beta 2, 3 and receptor 2 (*Tgfβ2, Tgfβ3, TgfβRII*)	Growth factor	*Tgfβ2^–/–^* and *Tgfβ3^–/–^* mice are born with congenital cyanosis and die within minutes (*Tgfβ2^–/–^*) or 24 h. after birth (*Tgfβ3^–/–^*). Double knockouts died around E15.5. and no tendons were detected. Ligaments in the limbs, trunk, tail, and head were missing.	Loeys-Dietz syndrome (TgfβRII-mutation)	[16,20,140,141]
Thrombospondin 2 (*Thbs-2*)	Glycoprotein	Postnatal (up to P4). Connective tissue, tail tendon, and invertebral ligaments present abnormalities associated with disordered collagen fibrilogenesis. *Thbs2*-deficient mice have hyperflexible tendons. Hind limb flexor tendons show less organization in fiber-forming compartments and larger fibroblast-defined compartments. Orientation of the fibers and fibril packing within the fibers are less regular. Collagen fibrils present larger diameter and uneven contours. *Thbs2*-deficient mice show difficulties in pulling their body up to the base of tail.	None reported	[142,143]
Thrombospondin 4 (*Thbs-4*)	Glycoprotein	Postnatal. In *Thbs4*-deficient mice, collagen fibrils and fibers in patellar tendons are larger compared to WT littermates. Nonexistent change in fibril shape. No compensatory overexpression of *Thbs-3* and *Thbs-5*.	SNPs associated with increased risk of premature myocardial infarction	[19,144–147]

ECM, extracellular matrix.

**Table 2.** Conditional Knockout and Reporter Mouse Models

*Mouse model*	*Mouse line*	*Description (what was the line used for?)*	*Reference*
Reporter	ScxGFP	Allowed the identification of Scx-expression sites and the study of tendon/ligament lineage.	[95]
Reporter	ScxAP	Reporter line facilitates the identification of tendon cells and phenotypic analysis in a wide range of genetic backgrounds	[95]
Conditional	ScxCre-L ScxCre-H	Transgenic mouse lines were used for targeting genes specifically in the Scx-expressing domain.	[66]
Conditional	Scx^Cre/Cre^ KI	Mouse line was used to identify that Scleraxis is transiently needed for proper tissue maturation and integration of musculoskeletal components.	[148]
Reporter	Col1a1GFP	Reporter mice were used for the identification of a cell subpopulation at different stages of skeletogenesis.	[149,150]
Conditional	Col5a1^flox/flox^	Mice strain was crossed with ScxCre transgenic mice for the generation of a tendon and ligament-specific collagen V-null mice.	[151]
Reporter	Nes-GFP	GFP expression was used to isolate a subpopulation of nestin^+^ TSPCs. After single-cell analysis, gene expression profiles revealed that nestin expression was activated at specific stages of tendon development. Isolated nestin^+^ TSPCs showed superior tenogenic capacity.	[152]
Conditional	Dcn^flox/flox^/Bgn^flox/flox^	Used to study the role of both proteoglycans in mature tendons. Tendons showed alterations in collagen fibril structure, realignment, and mechanical properties.	[153]

## Open Questions, Concluding Remarks, and Future Perspectives

The goal of this review was to transmit in a comprehensive manner how important basic research has been and still is for the appropriate understanding of tendon tissue, its biology, development, and function. It is remarkable that even though ∼45% of all musculoskeletal injuries in the United States each year are due to tendons and ligaments [89,92] very little attention has been given to the study of these tissues. The shortage of unambiguous tendon markers and the absence of suitable cell lines has been a constant issue impeding the breakthrough needed for understanding the mechanisms behind tendon development, maintenance, and repair. Basic research using small animal models (mouse and rat models) and knockout technologies has proven to be a success for the study of single genes, their function and role within a specific system.

On one side, the Cre/loxP- and Flp/FRT- technologies represent a valuable and powerful tool for the study of single genes in a controlled context and tissue that needs to be further investigated. Initially, the expression of Cre and Flp was not expected to be harmful because it was thought that mouse genome would not contain endogenous loxP or FRT sites [93] but Thyagarajan et al. showed in vitro, that sequences from human and mouse genomes appeared to be different from loxP and could support Cre-mediated recombination, thus, resulting in reduced specificity [94].

The possibility of combining ScxCre transgenic mouse line [66] with floxed genes, or the generation of new Cre-lines under the control of known tendon-related genes (Tenomodulin-Cre, Mohawk-Cre, Collagen type I-Cre, and Thrombospondin-Cre) would contribute immensely to augment our knowledge on the field and would allow us to obtain more information about signaling pathways, and upstream and downstream molecular regulators. On the other side, reporter lines such as ScxGFP [95] have already helped to decipher the pivotal role of the master transcription factor Scleraxis for tenogenesis and tenogenic differentiation. Following this example, it would be of importance to continue the efforts generating transgenic KO mouse models that mimic mutations and diseases observed in humans and to improve our understanding on the molecular mechanisms behind such conditions.

The most important and remaining challenge in the field is related to finding specific tendon markers at each developmental stage. This knowledge would not only contribute to the obtention of valuable information but also opens the possibility for the production and generation of drugs that could “boost” tendon healing.
